# Action of the Second Class of Voluntary Muscles

**Published:** 1862-06

**Authors:** 


### THE ACTION OF THE SECOND CLASS OF VOLUNTARY MUSCLES,

*That they are attached at both extremities; but their action is that of extension and retraction, simply.*

CONTINUED FROM PAGE 293.

84. For the plainest instances of this action, we must again have recourse to some of the inferior orders of animals. The land Terrapin furnishes several instances of this character, in the protrusion of its head, in the opening of its valves, &c.

From that part of the interior of the Carapax or top shell that corresponds to the lumbar vertebræ. Each of these muscles has three distinct heads or bundles of longitudal fibres, the longest of which arises from the most posterior vertebræ, and extending along on the side of the dorsal and cervical vertebræ, is inserted into the Occiput; the other two bundles of fibres arise a little in advance of this, and are inserted into the two cervical vertebræ nearest the head. Any one, who understands this arrangement of the muscles, and who has become acquainted with our view of muscular action, will readily comprehend how the head of the Terrapin is protruded, simply by the inervation and consequent elongation of these muscles; and he will readily comprehend how the head, when protruded, can be retracted within the shell, by the withdrawal of the nerve-fluid from these muscles.

85. In order to protrude the head, the Terrapin must first open its shells for this purpose. This it does by pushing down the anterior valve of the Plastrum or bottom shell, by which the space between the shells is closed. To enable it to do this, it is provided with two muscles, arising one on each side of the anterior dorsal vertebræ, which, passing down on either side of the neck, are inserted into the front edge of the anterior valve. By elongating these two muscles the valve is pushed down and the shells opened. The limbs of this animal are protruded and retracted by a similar arrangement of muscles, destined to this end.

86. The Snail (Helix nemoralis) presents some remarkable instances of the action of muscles that belong to this class. The soft parts of this animal consist mostly in a large bundle or mass of longitudinal muscular fibres, with which the several parts, as the head, the foot, &c., are protruded from the helix or turbinated shell; that is, by the active extension of these fibres.

The eyes of the Snail may be observed, when the animal is in motion, at the extremities of two fleshy tubes, or horns as they are commonly called, projecting from the head. From the mass of longitudinal fibres, two separate bundles or distinct muscles arise, and passing through the visceral cavity and traversing the horns, are inserted, one around the base of each eye, at the extremities of these horns. When the Snail is at rest, the Opthalmic muscles are retracted, the horns inverted, and the eyes are thus securely packed away in the visceral cavity; but when it is aroused, and protrudes its head and foot, it also determines its nerve-fluid to the Opthalmic muscles, and by elongating them, pushes forth its eyes. The horns are tubes filled out with muscular fibres placed spirally about the walls; and when the longitudinal muscles are extended these fibres are also extended; and in this way the calibres of the horns are enlarged, and thus allow a free action of the internal muscles with their nerves and blood-vessels.

It will be observed that there is a striking resemblance in the arrangement and action of the muscles in this instance to those in the instance formerly mentioned of the tongue of the Ant-eater. The explanations given by Physiologists of these two phenomena are the same, and both are equally absurd. They say, the eye of the Snail is everted by the contraction of the spiral muscle of the horn! The longitudinal muscle, they suppose, is intended and used solely for the purpose of retracting the eye!

87. We will advert to but one other instance of the action of the muscles of this class; it is to that of the muscles of the body of the Leech. This animal is possessed of two fleshy discs, one at each extrimity of the body, by means of which it attaches itself to the surface on which it crawls; the discs acting as cupping-glasses, and taking a firm hold wherever applied.

The walls of the body of the Leech are mainly composed of muscular fibres, that are arranged in three distinct layers. The fibres of the outer layer are disposed circularly, those of the middle layer spirally; but the fibres of the internal layer are all arranged longitudinally. By means of the later layer of fibres the progression of the leech is effectual. When stationery, the discs are attached to a surface near each other; but when about to move forward, the leech first detaches the anterior disc, and then extending its body by elongating the longitudinal muscles, it again attaches this disc to the surface on which it is crawling, or to some near object. When this anterior disc is fixed, the posterior one is detached and brought up, by the retraction or contraction of the same muscles, to be attached again near the former. In this manner the leech moves from point to point in its progress, sometimes with considerable celerity. In the same manner, the progression of all crawling animals, Annelidans, Serpents, &c., is effected. They first fix some part of the body, and from this point the body is extended by the elongation of the longitudinal muscles, when they again fix the forward portion of the body, and by contracting these same muscles by withdrawing the nerve-fluid from them, they draw up their length from behind.

But why do I say this of *crawling* animals? when the same mode of progressing is common to *all* animals; as will be explained further on.

ACTION OF THE THIRD CLASS OF VOLUNTARY MUSCLES.

88. The sphincters or sphincter muscle that belong to this class are composed of fibres arranged circularly around the openings about which they are placed, as the Orbiculares palpebrarum, the Orbicularis oris, &c. In a state of rest or of repose these muscles are moderately contracted, and the openings are closed: they may be more forcibly contracted and the openings more firmly closed by an intentional, persistent withdrawal of the nerve-fluid from their fibres. But when it is designated to bring into use the eyes or the mouth, the sphincter muscles are innervated, their fibres become actively elongated, and the openings are dilated or expanded.

The error that has prevailed in relation to the action of these muscles has arisen from the difficulty in distinguishing the consciousness that attends the innervation of the muscles from that which attends the withdrawal of the nerve-fluid from them. The early physiologists mistook the one consciousness for the other; and this mistake has continued in the books to the present time. They supposed that inervation was attended with the active contraction of the muscles and the closing or shutting of the openings. The really active state of the muscles they called relaxation, and supposed it was the result of inaction, or of an absence of the cause of action.

89. The instances of the action of the voluntary muscles we have already presented, are so plain, and the explanations given of the phenomena attending such action are so simple, intelligible, and rational, that, it appears to me, they must produce in every well ordered, unsophisticated mind that considers them, a full conviction of the truth of the law of muscular action that we have suggested.

The absurdity of former explanations of some of the above phenomena we have pointed out in passing; but there are, besides, explanatious of others of these phenomena, given in accordance with the old theory, equally absurd and unphilosophical to which we have not adverted. The explanations to which we allude are founded in sophistry, and are clearly traceable to this source.

Finding it utterly impossible to furnish a rational explanation, with the received theory, of phenomena such as are presented in the protrusion of the tongue of the Serpent, above referred to, the Sophist, to get around this new difficulty, *invented a new term*, by which all the facts of the case are obscured, covered up, and ignored. The organs, the action of which he could not explain, which are said to be composed of *erectile tissue.* He did not stop to demonstrate even to himself this particular tissue, which in reality has no existence, (for these organs, like all the other soft parts of the animal body, are possessed only of the cellular, the muscular, and the nervous tissues.) This was no part of his design, his sole object being accomplished by the invention of the *term* that would serve, as it has served, to deceive the unwary and unreflecting. It is thought by these, to be a sufficient explanation of all such phenomena to say, that the organs concerned, are composed of erectile tissue, and become erected when actively elongated.

The idea commonly attached to this state of these organs is, that they are injected with blood, and are in this way extended. The fallacy of this idea is easily shown, however, by calling to mind the facts, that these organs are elongated before receiving their extra supply of blood; and that, in many of these instances, the celerity of the motion of the organ, as in the case before us, entirely precludes this idea. The blood could not, by any possibility, be transmitted through the vessels, with a velocity to correspond with the motion of the tongue of the serpent, as indicated above.

ACTION OF THE FOURTH CLASS OF VOLUNTARY MUSCLES,

*That are attached at both extremities, but make use of the bones as levers.*

90. It is more difficult to convey a just conception of the action of this than that of any other class of voluntary muscles; because the means employed to produce the results are, here, much more complicated. The bones of the limbs to which these muscles are attached, and which are made use of as levers, are provided with two distinct sets of muscles, one on each of the two surfaces towards their line of motion; and their motion is not produced by the action of either set of muscles exclusively, but is caused by forces exerted by both sets at the same time. I repeat, the movement of the limbs of animals is not due exclusively to the action of either of the two sets of muscles with which they are supplied, but each movement is effected by the action or active elongation of one set, and by the contraction of the opposing set.

91. Before entering upon the explanation of the action of this class of muscles, it will be well to call to mind three important truths, that are essential to a clear understanding of this action. These are, First, that the action and the contraction of a muscle are both vital phenomena, to be met with only in the living body, and are not represented elsewhere in any part of the ecomomy of nature. These states of the muscle in the living body are *sui generis*, and no proper conception of them can be gained by comparing them to those of a cord or pulley of any kind or of any condition, whether as wet or dry, elastic or inelastic, &c. Second, that the contraction of a muscle is as much the result of the operation of a law of nature depending on the presence of, or the determination to it, of this fluid; and Third, that the operation of a law of nature is always attended with force or power, as well as its action or active elongation.

Force, it must be admited, is exerted in connection with the contraction of a muscle; but it is all important that we should not associate in our minds the force, with this state of the muscle, and then call this state of contraction the state of action of the muscle. This is the grand mistake committed by Physiologists, that has led them to overlook entirely the true state of action of a muscle, together with the force that is also exerted in connection with this state; and this mistake has led to their confusion of thought, and defective and erroneous views on the subject of muscular action.

I must repeat, the active state, or the state of action of a muscle, is a state of active elongation of its fibres; and in connection with this state, force or power is always exhibited.

92. With the knowledge of the above truths, the explanations we proceed to give of the action of this class of muscles will be rendered intelligible. The instances we shall select for this purpose will be such as are plainest and most familiar; that is, those that are to be observed in the action of the muscles of the limbs, upper and lower, of the human subject.

The action of these muscles are so misunderstood by physiologists, and so misrepresented in the books, that the technical terms employed to designate them are calculated to mislead. I shall, therefore, discard these terms as much as possible from our present consideration, and substitute others. In doing this, let it be understood, that those I propose are suggested only for a temporary use—to simplify the subject to the general reader, and to elucidate the principles involved. I hope, however, that some new terms for these muscles may be permanently adopted that will be more expressive of the true facts of the case than those now employed.

93. The muscles on the anterior surface of the Humerus or bone of the upper arm, embracing the Biceps and Brachialis anticus, we shall regard as one muscle, and call it, from its position, the Præ-humeral muscle; and that on the posterior surface of this bone, the triceps, we shall designate as the Post-humeral muscle.

The Præ-humeral muscle, then, arises from the shoulder blade (scapula) near the joint of the shoulder, and from the bone of the upper arm, and passing along its anterior surface is inserted into the upper portion of the bones of the fore arm.

The Post-humeral muscle arises also in part from the scapula, and in part from the humerus, and passing along the posterior surface of this bone is inserted into a process of one of the bones of the fore arm, called the Olecranon process, that projects behind the joint at the elbow.

94. When the whole arm is extended or straightened out from the flexed position, the mode in which this extension is effected by means of the muscles just mentioned, after what has been said above, is plain and palpable. In this movement, the anterior or Præ-humeral muscle is actively elongated by the determination to it of the nerve-fluid; and at the same time the posterior or Post-humeral muscle is contracted by having the nerve-fluid withdrawn. The motion, it will be observed, is produced by both muscles, the anterior and the posterior; but the former only is in action, or is influenced by the cause of action—the nerve fluid—it is in a state of vital erection; while the latter is thrown into a peculiar condition, that of contraction, by the abstraction of the cause of action. Both of these conditions, that of active elongation and that of contraction, in the opposing muscles, are essential and indispensable in every movement of the limbs, and each of these conditions being the result, as we have seen, (87) of the operation of a law of nature, such movements are executed with a double force, or with two forces, one of which is derived from the operation of each of these laws of nature.

The opposite movement of the arm—the flexing it at the elbow from the straight position—is produced by an opposite condition of the muscles engaged. In this movement, the posterior muscle is innervated and elongated, while the anterior muscle is contracted. This action of the posterior muscle is better shown, however, in the following instance:

95. The action of the muscles by means of which the fingers are moved, will be best shown by regarding them as two muscles, one on each surface of the bones of the fore arm, from which they arise. We will call them for the present, the Præ-brachial and the Post-brachial muscle, each term embracing the long muscles that go to the several phalanges of the fingers on its respective surface.

The anterior or Præ-brachial muscle arises from the bones of the fore arm towards the elbow, on the inner surface, and extending along in front, passes with its long tendons across the palm of the hand, to be inserted into the several bones of the phalanges of the fingers on their inner surface.

The posterior or Post-brachial muscle in like manner arises from the upper portion of the same bones, but from their outer surface, and passing along on this surface, and with their tendons over the back of the hand, are inserted into the bones of the phalanges of the fingers on their outer surface, extending along even to the extremities of these bones.

96. The great difficulty in realizing the true action and agency of the muscles we have just spoken of, arises from the fact mentioned above, when speaking of the sphincters; namely, that few minds are capable of distinguishing the consciousness or the mental sensation that attends the determination of the nerve-fluid to a muscle, that causes its action, from the consciousness or sensation that attends the abstraction of this fluid, that causes the contraction of a muscle. To make this distinction, it requires a patient education and training of the mind, more especially in those who have given some attention to physiology, and who have their minds pre-occupied with erroneous views of muscular action. This mental preparation, however, is what few or none have set about; but when this difficult task is accomplished, it will be observed, that,

97. In flexing the hand, as in forcibly shutting it, or in grasping any object firmly, a determination of the nerve-fluid is made to the posterior or Post-brachial muscle; and its fibres becoming actively or forcibly elongated, tend to produce the movement indicated; at the same time, the nerve-fluid is withdrawn from the anterior or Præ-brachial muscle, and its fibres becoming forcibly contracted, the act of shutting the hand, or of grasping, is perfected and completed.

The opposite states of these muscles—that is, the innervation and extension of the anterior, and the enervation and contraction of the posterior muscle—it is evident, would produce the contrary movement of the fingers, the extending or straightening them out.

98. If I have succeeded in explaining to the satisfaction of the reader, the action and agency of the muscles engaged in effecting the movements of the upper extremities, there will be no difficulty whatever in his comprehending the action and agency of the other muscles of this class that are concerned in the various voluntary movements of the living body. In all such movements, we repeat, two sets of muscles are concerned, whose action is opposed, the one to the other; but the position established is the result of the agency, mainly of the set that is in a state of action, although in some measure attributable to the contraction of the opposing set. Thus, in assuming and maintaining the erect position of the body, the muscles, on the anterior surfaces of the lower extremities, of the spinal columns, and of the walls of the abdomen and thorax, are brought into action, and the result is accomplished, principally by the agency of these muscles, but in some measure also, by the contraction of those on the opposite surfaces. Again:

99. In locomotion, of the biped, for example, it is true, the hinder foot is brought up to the advanced position; and, again, the foot is raised to be carried forward by the contraction of one set of muscles; but the progressive motion—the extending the leg and foot in advance, and the projecting forward of the whole body—is effected by the action, the active elongation, or extension of the other set; or the opposite conditions of the same muscles may be employed for both of these purposes.

The mode of progression, we have said, (87) is the same in all animals. In all, a portion of the body is advanced from a fixed point by the action or extension of certain muscles, and in all, a portion of the body that is behind this point is brought up by the contraction of certain muscles. In this respect the halting gait of the biped—that is to say, when, in walking, he keeps the same foot always in advance, bringing up the hinder foot to it, and then moving the front foot forward again—is the same with the natural gait of the snail; and, again, the snail would imitate the natural gait, or the continued progressive motion of the biped, if, instead of attaching its hinder disc in the rear of the front one, it could carry it along forward, and attach it in an advanced position, and so continue its motion, carrying its discs alternately forward.

100. The arrangement of the muscles, however, in the lower extermities is somewhat different from that of the upper. The extension of the leg on the thigh is effected by the action of the muscles on the posterior surface of the thigh bone or femur, while the leg is flexed on the thigh by the action of the muscles on the anterior surface of this bone; and, again, the action of the tibialis anticus, and of the anterior longus digitorum on the anterior surface of the leg or tibia, tends to extend or straighten out the foot on the leg; and of the latter, to flex the toes on the sole of the foot; while the action of the muscles on the posterior surface, the Gastrocnemius, the Soleus, and the posterior longus digitorum, &c., tends to flex the foot upon the leg anteriorly at the ankle—that of the two former by pressing down the os calcis as a lever, and of the latter by extending and directly raising the toes.

A curious circumstance, confirmatory of the correctness of this view of the action of the muscles of this region, is mentioned by Mr. John Hunter, who suffered from a fracture of the tendon of the gastrocnemius, called the tendo Achillis. When this fracture occurred, he experienced, he says, the greatest difficulty in raising the toes from the floor, in walking across the room.

Nothing has been said of the contraction of the opposing sets of muscles which assists materially in the movements just mentioned, because that will, of course, be inferred from what had been said above; and to avoid encumbering the subject, relating to the physiological principle here advocated, we have omitted altogether the consideration of the mechanical principles of the Lever and Pulley that are involved in all the movements of the extremities.

